# Eco-Design of Thermopressing through Induction of 100% Coriander-Based Fiberboards: Optimization of Molding Conditions

**DOI:** 10.3390/ma17194852

**Published:** 2024-10-01

**Authors:** Priscila Guaygua-Amaguaña, Guadalupe Vaca-Medina, Claire Vialle, Caroline Sablayrolles, Philippe Evon

**Affiliations:** Laboratoire de Chimie Agro-industrielle, INRAE, INPT, Université de Toulouse, 4 Allé Emile Monso, CS 44362, 31030 Toulouse, France; guadalupe.vacamedina@toulouse-inp.fr (G.V.-M.); claire.vialle@ensiacet.fr (C.V.); caroline.sablayrolles@ensiacet.fr (C.S.)

**Keywords:** plant-based fibers, thermopressing, induction, fiberboards, mechanical properties, climate change

## Abstract

The hot pressing process for 100% coriander-based fiberboards was optimized using an induction RocTool system, which offers rapid mold heating and cooling. The fiberboards were made using deoiled press cake as a protein binder and extrusion-refined straw as reinforcement. Doehlert’s experimental design was used to evaluate the influence of pressure (10–50 MPa), molding time (60–300 s), and mold temperature (155–205 °C) on fiberboard properties, energy consumption, cost, and environmental impact. The results showed that the RocTool device allows for better temperature control during shaping throughout the mold, resulting in mechanical properties that are both more homogeneous across the entire surface of the panel and, more importantly, substantially improved. Using the isoresponse curves, the optimal hot pressing conditions were 35 MPa, 300 s, and 205 °C, corresponding to a 40.6 MPa flexural strength. However, it was observed that to achieve an MDF-like fiberboard with minimal production costs, much less restrictive molding conditions were sufficient, i.e., 32.5 MPa, 170 s, and 160 °C. The study revealed that maximum thermopressing conditions emitted 3.87 kg of CO_2 eq_., while conditions leading to the MDF-like board reduced emissions to 1.45 kg CO_2 eq_., resulting in a more environmentally friendly material.

## 1. Introduction

The construction industry within the European Union (EU) is a major consumer of both biotic and abiotic resources, using about 50% of the materials extracted in Europe. Building materials alone are responsible for emitting approximately 250 million tons of CO_2_ annually. The industry accounts for 33% of freshwater consumption and generates 35% of the total waste in the EU. Globally, the sector is responsible for 53% of greenhouse gas emissions and 60% of energy use [1]. A substantial portion, around 70%, of wood in the EU is used in construction and furnishing applications [1]. Despite wood’s reputation for having a lower carbon footprint compared to other construction materials, concerns remain regarding its sourcing [2]. Activities such as inadequate forest management and unauthorized tree removal can have adverse effects on both local populations and ecosystems, leading to a decline in biodiversity. Furthermore, unsustainable practices like illicit logging contribute to broader environmental issues such as deforestation, soil erosion, droughts, wildfires, and desertification [3].

Given the contracting market for wood fibers, there has been an increasing interest for many years in exploring plant-based fibers as a cost-effective and widely available alternative [4]. In pursuit of better resource management solutions, the EU promotes the use of bio-based products through its Bioeconomy Strategy [5]. This strategy aims to reduce dependence on non-renewable resources while mitigating and adapting to climate change. However, any alternative products must match traditional non-renewable and wooden materials in terms of usage properties, durability, and cost competitiveness.

Over the past few years, various bio-based boards have been successfully manufactured using (thermo)pressing techniques, demonstrating good performance. These boards, made from corn stalks [6], wheat straw [7], rice straw [8], flax and hemp bast fibers [9], sunflower stalks [10], coriander straw [11], sugarcane bagasse [12], miscanthus [13], cotton stalks [14], date palm biomass [15], and oil palm wastes [16], exhibited interesting mechanical and water-resistance properties. For example, corn stalks combined with Kraft lignin produced high-density fiberboards (HDFs) with enhanced modulus of rupture (MOR), and modulus of elasticity (MOE) [6]. Wheat straw, bonded with melamine-modified urea–formaldehyde resin, resulted in medium-density fiberboards (MDFs) with a MOR ranging from 23 to 37 MPa [7]. Rice straw panels, bonded with urea–formaldehyde resin, had MOR values exceeding 26 MPa, an MOE above 3.1 GPa, and less than 12% thickness swelling, meeting MDF standards [8]. Flax and hemp fibers significantly increased the MOR and MOE of particleboards by up to 60% and 46%, respectively [9]. Sunflower stalks, when combined with chitosan, formed insulating bio-composites with thermal conductivities between 56 and 58 mW/m K [10]. Cotton stalks, using urea–formaldehyde resin [14], and palm biomasses [15,16], with phenol–formaldehyde resin, also demonstrated strong performance as alternative raw materials for particleboards. In binder-free applications, sugarcane bagasse was self-bonded [12], while miscanthus stalks produced particleboards with MOR and MOE slightly lower than wood-based boards, though still suitable for general use in dry conditions [13].

In particular, recent research has demonstrated the feasibility of using straw from the coriander plant and fruit press cake to produce renewable boards with low volatile organic compound (VOC) emissions [11]. These boards emerge as viable contenders to traditional wood-based panels, offering affordability, attractive flexural properties, and a straightforward manufacturing process.

Coriander (*Coriandrum sativum* L.), globally recognized for its culinary and medicinal uses, is an annual herb belonging to the Apiaceae family, with a global production estimated at 600,000 tons annually [17]. Its seeds, which contain both vegetable oil and essential oil fractions, have gained renewed attention in recent years. Coriander vegetable oil is particularly notable for its high content of petroselinic acid, an isomer of oleic acid, comprising up to 75% of its fatty acid profile. This acid has sparked interest across food, cosmetics, and pharmaceutical industries and is poised to play a pivotal role in oleochemistry for synthesizing diverse platform molecules in the near future [18]. Moreover, the vegetable oil extracted from coriander seeds was certified as a Novel Food Ingredient by the European Food Safety Authority in 2013 [19], allowing for its use as a dietary supplement.

Despite being considered crop residue, coriander straw constitutes up to 80% (*w*/*w*) of the plant’s aerial biomass, offering a low-cost resource priced at approximately EUR 90 per ton. Currently, around 250 tons of coriander straw are available annually in southern France. With the growing popularity of coriander vegetable oil, an increase in straw availability is anticipated in the coming years [18].

The press cake obtained through mechanical treatment of the seeds in a twin-screw extruder contains proteins and lignocellulosic fibers [11]. These proteins can act as natural binders in boards molded through hot pressing, eliminating the need for synthetic binders such as formaldehyde-based thermosetting resins to produce cohesive panels. Simultaneously, lignocellulosic fibers enhance the mechanical strength of the boards, while straw provides structural support as a mechanical reinforcement [20]. Therefore, straw and press cake derived from coriander seeds are two co-products of growing interest as materials for the construction sector.

This study aims to optimize the production process of coriander fiberboards by using an induction RocTool device, which employs high-frequency currents to generate heat in a tool through localized magnetic fields [21]. Known for its rapid mold heating and cooling capabilities, this system minimizes defects. Additionally, this approach improves the mechanical properties of the fiberboards, such as mechanical strength and water resistance, resulting in more homogeneous agromaterials. This equipment has very recently proven its efficiency on fiberboards made from sugarcane bagasse [22] or from brown seaweed *Sargassum* spp. [23].

Here, the goal is to manufacture fiberboards entirely derived from coriander, utilizing extrusion-refined straw as fibrous reinforcement and 40% (*w*/*w*) deoiled press cake as a protein binder. A Doehlert’s experimental design with three variables was carried out to evaluate the influence of thermopressing conditions (i.e., applied pressure, molding time, and mold temperature) on the 100% coriander-based fiberboard properties. Moreover, from this, optimal conditions were determined to produce a coriander material with maximal flexural properties. Additionally, the operational conditions for which the board’s flexural strength is equivalent to that of a commercial MDF panel while minimizing energy consumption were also identified.

## 2. Materials and Methods

### 2.1. Thermopressing of Fiberboards through Experimental Design

Fiberboards were produced using a 50-ton Pinette Emidecau Industries (Chalon-sur-Saône, France) heated hydraulic press and a 70 mm × 70 mm square mold. The 100% coriander-based raw material used in the study consisted of a premix made of an extrusion-refined coriander straw and a deoiled press cake in a mass ratio of 100 g:40 g. A continuous twin-screw extrusion process allowed for premix production.

For the thermopressing process optimization using the RocTool system (Pinette Emidecau, Charlon-sur-Saône, France), 20 g of premix (thus comprising 14.3 g of refined straw and 5.7 g of deoiled press cake) with 7.8% in moisture was used for each fiberboard. The variables studied were the applied pressure (from 10 to 50 MPa), the molding time (from 60 to 300 s), and the mold temperature (from 155 to 205 °C). The RocTool system maintained mold temperature accuracy within ±2 °C of the set point, ensuring consistent temperature across the fiberboards during thermopressing. In addition, it is important to note that (i) the glass transition temperature (T*_g_*) of coriander protein was evaluated at 140–145 °C using differential scanning calorimetry (DSC) technique, and (ii) the thermogravimetric analysis (TGA) showed the onset of thermal degradation at 225 °C [24]. Therefore, by working between 155 °C and 205 °C, we can ensure that the proteins transition into a rubbery, viscous state during shaping without undergoing thermal decomposition.

A Doehlert design matrix was implemented to optimize the process. It is a statistical experimental design method used for optimizing processes or products by studying multiple factors simultaneously within a minimum of experiments. The matrix used a quadratic polynomial model to describe the relationship between the experimental variables and the responses [25]:

(1) $$y=b_{0}+\sum_{i=1}^{3}b_{i}x_{i}+\sum_{i=1}^{3}b_{ii}x_{i}^{2}+\sum_{\begin{array}{c} i=1\\ i\neq j \end{array}}^{3}\sum_{j=2}^{3}b_{ij}x_{i}x_{j}$$

where $x_{i}$ and $x_{j}$ are the coded values for the independent variables, ranging from −1.0 to 1.0, and $b_{0},b_{i},b_{ij}$ are the regression coefficients obtain through regression analysis. The number of levels for the three studied variables were 5 levels for the applied pressure (*x*_1_), 7 levels for the molding time (*x*_2_), and 3 levels for the mold temperature (*x*_3_). The central point of the experimental matrix was replicated four times, resulting in a total of 16 trials. All of the fiberboards were then characterized. Table 1 shows the experimental matrix used in detail. The experimental data for the responses (*y*) (i.e., density, flexural properties, color characteristics, surface hardness, cost-related factors, and environmental indicator) were modeled using the NemrodW software, v2000 [26].

### 2.2. Board’s Characterization

#### 2.2.1. Density

The manufactured fiberboards were cut into seven test specimens with dimensions of 10 mm in width and 50 mm in length. Their thickness (t, mm) was approximately 3 mm, slightly varying based on the hot pressing conditions applied. Before the characterization, specimens were equilibrated in a climatic chamber at 50% relative humidity and 25 °C until they reached a constant weight.

The apparent density of each specimen was determined by weighing it on a Sartorius precision balance with a precision of 0.01 mg (Goettingen, Germany) and by measuring its dimensions with a sliding caliper with a precision of 0.01 mm. Measurements were taken at three different points along each specimen for thickness and width and at two points for length. The volume of each specimen was then calculated using the average values of these measurements. Finally, the density was calculated by dividing the mass by the measured volume, and it was expressed in kg per m^3^. The density measurement was repeated 7 times for each fiberboard, using the seven previously cut test specimens.

#### 2.2.2. Flexural Properties

The flexural properties of the fiberboards were determined according to the ISO 16978:2003 standard [27]. The three-point flexural tests were conducted with a Tinius Olsen H5KT Benchtop Materials Testing Machine (San Diego, CA, USA) fitted with a 5 kN force sensor. A distance of 40 mm was employed, along with a testing speed of 1 mm/min. The flexural properties determined were the breaking force (F_max_, N), the flexural strength (σ_f_, MPa), and the flexural modulus of elasticity (E_f_, GPa). The flexural test was repeated 7 times for each fiberboard.

#### 2.2.3. Shore D Surface Hardness

The determination of indentation hardness by means of a durometer of the coriander fiberboards was performed, conforming to the ISO 868:2003 standard [28]. The durometer used was a Bareiss Shore D (Oberdischingen, Germany). The tests were performed on the lower and upper surfaces of the fiberboard (5 measurements on each side of each test specimen), and this resulted in 70 replications for each fiberboard.

#### 2.2.4. Internal Bond Strength

Measurements of internal bond strength were conducted only on the optimal and MDF-like fiberboards from 50 mm × 50 mm square specimens. An Instron 33R 4204 (Norwood, MA, USA) universal testing system and the ISO 16260:2016 standard [29] were used. Analyses comprised four repetitions for each material tested.

#### 2.2.5. Color Characteristics

The color characteristics were determined with a Konica Minolta CR 410 (Tokyo, Japan) colorimeter, provided with pulsed xenon arc lamp and 6 silicon photocells. Results were expressed in the CIE L*a*b* color space referential defined by *the Commission Internationale de l’Eclairage* (CIE), where color is expressed as three values. The lightness value, L*, defines black at 0 and white at 100. The a* axis relates to green–red opponent colors, ranging from negative values for green to positive values for red. Conversely, the b* axis represents blue–yellow opponents, with negative values indicating blue and positive values indicating yellow.

Additionally, the color difference (Δ*E*) between the starting premix material and the fiberboard was calculated according to the ISO/CIE 11664-4 standard [30].

(2) $$\Delta E=[({L_{1}}^{*}-L_{0}^{*}{)}^{2}+(a_{1}^{*}-a_{0}^{*}{)}^{2}+(b_{1}^{*}-b_{0}^{*}{)}^{2}{]}^{\frac{1}{2}}$$

where *L*_0_^*^, *a*_0_^*^, and *b*_0_^*^ are the color parameters of the starting premix material measured after very low pressing and without heat, and *L*_1_^*^, *a*_1_^*^, and *b*_1_^*^ are the board’s color parameters. Measurements were conducted on both sides of the fiberboard surface, totaling 18 measurements for each fiberboard.

#### 2.2.6. Water Sensitivity

Exposure to water can affect the properties of the materials. The determination of thickness swelling (TS, %) and water absorption (WA, %) of the coriander fiberboards was performed after 24 h immersion in water, according to the ISO 16983:2003 standard [31], where the three test samples used measured 50 mm × 50 mm. The test was conducted in a hydroclimatic room. The water absorption of the test samples was determined gravimetrically, and the thickness of the square specimens before and after water immersion was measured on each side using a sliding caliper with a precision of 0.01 mm.

### 2.3. Cost-Related Factors

#### 2.3.1. Energy Consumption

The energy consumption of the thermopressing process of the coriander fiberboards was estimated on the basis of the characteristics of a Pinette Emidecau Industries (France) 400 industrial-sized hydraulic hot-plate press that would allow for the manufacture of 280 mm square panels, including those molded at a maximum pressure of 50 MPa. It was then analyzed through the Doehlert experimental design presented in Table 1. The characteristics of the thermopressing machine are as follows:

- A closing force of 400 tons (corresponding to a 275 bars maximal hydraulic oil pressure in the piston).
- There was 33 kW for the available heating power (total for both hot plates) and 250 °C for maximum attainable temperature.
- There was 22 kW for the pump unit power required to close the press at 275 bars (maximum attainable pressure).

#### 2.3.2. Total Manufacturing Cost

The total manufacturing cost included the cost of raw materials (i.e., coriander straw and press cake from seeds), the production cost of the coriander premix in the twin-screw extruder, including the extrusion–refining of straw and its mixing with the press cake, and the cost of thermopressing. On the one hand, the two first costs were estimated to be 0.55 €/m^2^ and 0.83 €/m^2^, respectively. On the other hand, the cost of thermopressing was deduced from the estimated energy consumption during molding. The electricity price used for calculations was set at 0.25 €/kW h, corresponding to the average electricity price based on the energy mix in France in 2023.

### 2.4. Climate Change as Environmental Indicator

The determination of an environmental indicator included the life cycle assessment (LCA) of the production of the coriander fiberboards. This study followed the principles established in the ISO 14044:2006 standard [32]. The functional unit chosen was the production of 1 m^2^ of coriander fiberboard. To evaluate the environmental performance of the coriander fiberboard, a gate-to-gate approach LCA was carried out. The database used was Ecoinvent v3.9.1 Cut-off, and the scenarios were established according to the experimental design shown in Table 1 and analyzed with SimaPro 9.5.0.2 software [33]. To assess the impact, the energy consumption of the thermopressing process was evaluated. The background data used for this assessment were “Electricity, medium voltage {FR}|market for electricity, medium voltage|Cut-off, U”. The life cycle impact assessment results were evaluated using Environmental Footprint 3.1. In this study, the only impact category considered was climate change.

## 3. Results and Discussion

**Figure 1:**
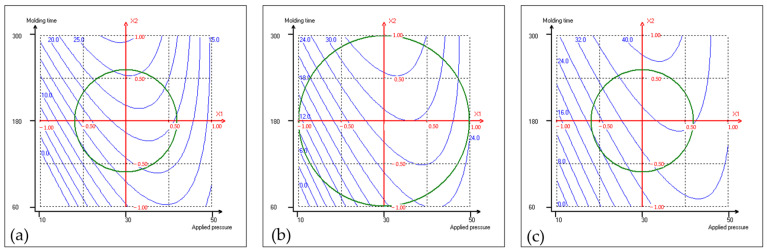
Isoresponse curves for flexural strength (MPa) at 160 °C (**a**), 180 °C (**b**), and 200 °C (**c**) mold temperatures.

**Figure 2:**
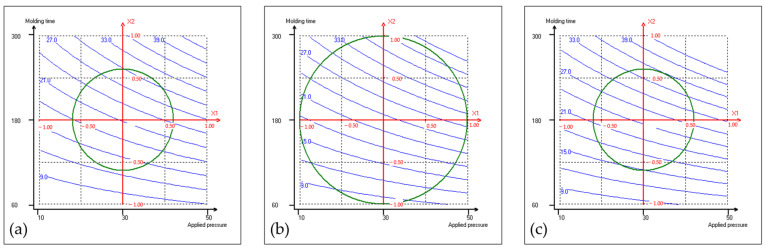
Isoresponse curves for energy consumption (kW h/m^2^) at 160 °C (**a**), 180 °C (**b**), and 200 °C (**c**) mold temperatures.

**Figure 3:**
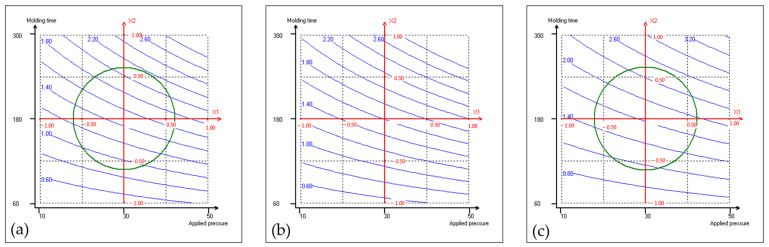
Isoresponse curves for climate change (kg CO_2 eq_/m^2^) at 160 °C (**a**), 180 °C (**b**), and 200 °C (**c**) mold temperatures.

The experimental design comprised 16 trials (Table 1). First, all of the obtained fiberboards were cohesive and machinable, demonstrating the adhesive effect of proteins derived from the press cake. The thickness, density, flexural properties, Shore D surface hardness, color characteristics, cost-related factors, and an environmental indicator of the resulting coriander boards were assessed, and they are presented in Table 2; the thickness of the fiberboards tested was around 3 mm. For each response, these results were used to build the mathematical model shown in Section 2.1. Quadratic polynomial equations were applied to the collected data, and the corresponding coefficients, along with the R^2^ values, were determined. They are presented in Table 3. Each response model exhibits an R^2^ value surpassing 0.85, signifying quite satisfactory concordance between the models and the experimental data points. Figure 1, Figure 2 and Figure 3 show the isoresponse curves for flexural strength, energy consumption and climate change at 160 °C, 180 °C, and 200 °C mold temperature, respectively.

Depending on the hot pressing conditions applied, the density of the produced coriander fiberboards ranged from 1122 to 1434 kg/m^3^, meaning that all boards could be considered as hardboards. According to the model coefficients (Table 3), the applied pressure has the greatest influence, followed by the molding time and the mold temperature. An increase in pressure leads to a rapid increase in density, which is advantageous for flexural properties, as higher-density fiberboards exhibit superior mechanical characteristics. This can be confirmed by board 7, which shows a high density as well as high flexural properties (Table 2). Observations show that fiberboards manufactured at low pressures deform and acquire a slight concave contraction when removed from the mold.

The flexural properties are represented by the breaking force ($F_{max}$), flexural strength ($\sigma_{f}$), and modulus of elasticity ($E_{f}$). The thermopressing conditions exert a strong influence on these properties, with values ranging from 13.2 to 46.2 N, 6.6 to 36.0 MPa, and 1.0 to 6.2 GPa, respectively. Unlike the density of the board, the paramount parameter is the mold temperature, especially for the breaking force and flexural strength, and, to a lesser extent, the molding time, while the applied pressure has a lower impact on these properties. Boards 6 and 7 highlighted the significance of mold temperature, as higher temperatures resulted in high flexural properties that were relatively close to each other.

The trend observed in the density is repeated in the Shore D surface hardness, where the applied pressure has the greatest impact, with values ranging from 65.4 to 78.4°. It is noted that board 2, with the lowest surface hardness, was also manufactured with the lowest pressure, thus confirming the previously proposed hypothesis.

The color characteristics indicate that the applied pressure has the greatest influence on this property, followed by the molding time and mold temperature. Observations revealed that as the lightness (L*) value decreases, the flexural strength of the fiberboard increases. Furthermore, the color difference (Δ*E*) is significantly affected by the applied pressure and, to a lesser extent, by the molding time and mold temperature, respectively. These results show that the observed darkening of the panels is more pronounced when restrictive thermopressing conditions are applied, generating materials with greater flexural strength. In other words, the board color, which is easy to assess immediately after hot pressing, serves as a good indicator of its bending properties. The darker the panel, the greater its mechanical strength. In previous studies, darkening of sunflower proteins was also evidenced for thermo-molded films produced using a sunflower protein isolate [34] or for injection-moldable composite materials made of sunflower oil cake [35]. This darkening highlighted good plasticization of proteins at the moment of molding. Similarly, during the production of binderless boards from a coriander cake, it was observed that the darkening of the protein fraction increased when the gap between their glass transition temperature and the hot pressing temperature widened [11]. In this study, with more restrictive thermopressing conditions, proteins in their rubbery state were less viscous at molding, resulting simultaneously in a more pronounced darkening, just as improved fiber wetting.

The water immersion tests revealed generally unfavorable results for thickness (TS) and water absorption (WA) after 24 h, with the lowest values being 55% (board 12) for TS and 78% (board 7) for WA (Table 2). In some cases, TS measurements were impossible due to specimen disintegration after prolonged water exposure. As a result, the mathematical models for water sensitivity could not be calculated, and these properties are not listed in Table 3.

It was, however, observed that the molding conditions, particularly temperature and applied pressure, significantly influenced the water sensitivity of the fiberboards. Boards manufactured at the highest temperatures and pressures, as well as those produced under moderately high conditions, showed improved TS performance. Specifically, boards 1, 12, 15, and 16, which had the lowest TS values among the nine boards for which this property could be measured, were produced at high pressures (30 MPa or 50 MPa), and high temperatures (180 °C or 200 °C). These conditions resulted in denser material, improving water resistance.

Table 3 highlights that, from a manufacturing cost perspective, molding time stands out as the primary parameter. Increasing this parameter rapidly escalates energy consumption, thereby driving up production costs. This insight aligns well with production goals, as shorter production cycles are generally preferred. Considering manufacturing costs, the recommendation is to prioritize increases in temperature first, followed by adjustments in pressure, and finally, changes to molding time. Consequently, it is advisable to adjust molding time last, after exploring different pressure and temperature configurations to achieve the desired fiberboard properties.

On a different note, raising the mold temperature correlates with an observed increase in flexural strength. However, this enhancement has minimal impact on fiberboard costs due to the smaller coefficient b_3_ in comparison to the coefficients b_1_ and especially b_2_ in the mathemathical model.

It is noteworthy to mention that Figure 1c demonstrates that at the 200 °C highest mold temperature tested, the potential for achieving high flexural strength is greater, with values reaching up to 40 MPa, significantly higher than the value obtained at 160 °C. Conversely, at a mold temperature of 180 °C, flexural strength does not exhibit such a significant increase. This underscores the importance of both mold temperature and molding time to maximize the coriander fiberboard properties, highlighting that a good combination of these two factors is required to achieve the target properties.

The insights gained from the mathematical model, as represented by the isoresponse curves in Figure 1, enabled us to identify the operating conditions necessary to maximize the flexural properties of the product. This is crucial for ensuring the quality and durability of the coriander fiberboard. The thermopressing conditions to obtain a fiberboard with the maximal flexural strength were an applied pressure of 35 MPa (*x*_1_: 0.25), a molding time of 300 s (*x*_2_: 1.0), and a mold temperature of 205 °C (*x*_3_: 1.0). With mold temperature and molding time pushed to the upper limit of the design matrix, the flexural properties were maximized. Additionally, the applied pressure is maintained relatively low, thereby facilitating the transition to a large-scale industrial process.

Coriander fiberboards were manufactured again under the previously mentioned thermopressing conditions to maximize their flexural properties and to ensure the accuracy and reliability of the mathematical model. Table 4 presents a comparison between the mathematical model and the experimental results for the tested fiberboards. The coriander fiberboards manufactured had a flexural strength of 40.6 MPa, which is significantly higher (+40%) than the value previously obtained with a classical thermopressing (without RocTool), i.e., 29.1 MPa [11], demonstrating that the RocTool system significantly improves the flexural properties of the obtained material. When comparing the flexural properties of the new coriander fiberboards to the previously optimal one manufactured without the RocTool system, the elastic modulus (E_f_) also increased significantly from 3.9 GPa [11] to 6.7 GPa, marking a 72% improvement in this characteristic. Comparing the results obtained experimentally with those predicted by the mathematical model for flexural properties, its accuracy closely aligns with the experimental outcomes (e.g., −4.5% for flexural strength), underscoring its predictive value in estimating fiberboard characteristics under various pressure, time, and temperature conditions that may not have been directly tested experimentally.

The internal bond strength of the coriander fiberboards optimized for maximal flexural strength was also measured. It was equal to 1.45 MPa, which is significantly higher than the values recommended by ISO 16895:2016 [36] (standard dedicated to the specifications for particleboards), whatever the type of application concerned (e.g., 1.20 MPa min in the most demanding conditions for boards, i.e., HDF-GP MR1 and MR2, with a thickness >2.5 mm and <4.0 mm).

The energy consumption isoresponse curves (Figure 2) illustrate how energy consumption varies under different thermopressing conditions, providing detailed insights into the energy efficiency of the analyzed system. Figure 2a, Figure 2b, and Figure 2c show energy consumption variations under constant mold temperatures of 160 °C, 180 °C, and 200 °C, respectively. Figure 2 indicates that increasing pressure results in a moderate increase in energy consumption (blue curve), as illustrated by the intermediate value for the corresponding b_1_ coefficient (Table 3). Oppositely, increasing molding time results in a rapid rise in energy consumption, regardless of the mold temperature. Lastly, comparing Figure 2a, Figure 2b, and Figure 2c, higher mold temperatures also increase energy consumption but to a much smaller extent than for the other two operating variables. Figure 2c shows that at 200 °C mold temperature, energy consumption can exceed 39 kW h/m^2^. The increase in energy consumption, particularly noticeable with extended molding times and, to a lesser extent, elevated applied pressure and then mold temperatures, directly escalates production costs, highlighting the economic impact of optimizing energy efficiency in the fiberboard manufacturing process.

It is crucial to remember that achieving coriander fiberboards with maximum properties involves increased energy and resource consumption (i.e., 43.1 kW h/m^2^, calculated on the basis of the mathematical model), resulting in elevated production cost (i.e., 12.51 €/m^2^). Nonetheless, a commercial medium-density fiberboard (MDF) has a flexural strength of 20.7 MPa [11]. So, the coriander fiberboard does not necessarily need to have a flexural strength as high as the one obtained under maximum conditions (40.6 MPa). Figure 1a shows that at mold temperature of 160 °C, it is possible to obtain the desired strength. Therefore, aiming to manufacture a coriander fiberboard with minimal energy consumption, guided by the isoresponse curve of energy consumption (Figure 2a), while meeting strength requirements for an MDF-like board, the two isoresponse curves (Figure 1a and Figure 2a) were juxtaposed to identify the most suitable conditions. The analysis showed that the minimum energy consumption to obtain 20.7 MPa of flexural strength was 21 kW h/m^2^ according to the mathematical model, and the thermopressing conditions to achieve such energy consumption were an applied pressure of 32.5 MPa (*x*_1_: 0.125), a molding time of 170 s (*x*_2_: −0.085), and a mold temperature of 160 °C (*x*_3_: −0.816).

Similarly, as with the previous conditions, the manufacturing of coriander fiberboards were repeated again under these new conditions. Subsequently, a flexural test was conducted on the new fiberboards, revealing a flexural strength of 17.9 MPa (Table 4), which is noticeably below the expected mathematical model result (i.e., −13.5%). This discrepancy may be attributed to the reduced accuracy of the mathematical model within this range of the experimental plan, resulting in experimental values deviating further from the model-predicted values. For the rest of the flexural properties of this MDF-like boards, the results showed a maximum breaking force of 28 N and an elastic modulus of 2.9 GPa. When comparing these values with the results from the mathematical models, it is evident that the experimental values under these specific thermopressing conditions are slightly different from the expected values. This suggests that the model may need refinement to address these minor discrepancies. The internal bond strength of the MDF-like boards was also measured. Equal to 0.35 MPa (Table 4), this corresponds to a significant reduction (i.e., −76%), compared to the value observed for the boards with maximal flexural strength.

Another factor to consider is the molding time used for manufacturing MDF-like boards. As mentioned earlier, increasing molding time can significantly enhance the flexural strength of fiberboards. This is evident from the results of the experimental design (Table 2), where board 10, which had quite similar thermopressing conditions as those of the MDF-like board, with the exception of molding time, which was significantly higher (applied pressure: 30 MPa, molding time: 249 s, and mold temperature: 160 °C), exhibited a flexural strength of 24.1 MPa. This value exceeds the flexural strength required for an MDF-like board. The key factor influencing this result appears to be the molding time. A shorter molding time may not allow for adequate protein mobilization during the process and their proper distribution on the surface of the fibers, resulting in fiberboards with lower flexural strength. Additionally, it is crucial to consider the molding temperature (160 °C), as it is near the glass transition temperature (162 °C) of the press cake [11]. The combination of these two low thermopressing conditions could prevent the proteins in the press cake from fully reaching their rubbery state, leading to incomplete fiber wetting. Consequently, this could result in suboptimal mechanical properties. Therefore, it is recommended to increase the molding time to enhance the flexural strength of the MDF-like fiberboard.

The water sensitivity of the manufactured fiberboards was tested after 24 h of immersion in water. The results presented in Table 4 indicate that the coriander fiberboard, which demonstrated maximal flexural performance, exhibited a TS of 161% and a WA of 123%. In contrast, the MDF-like board showed a significantly higher WA of 198%, but its TS value could not be measured due to complete disintegration after immersion in water, with fibers separating from each other. As mentioned previously, water sensitivity properties improve under higher pressure and temperature conditions. The conditions used to achieve maximal flexural strength, which involved the highest pressure and temperature, increased the water resistance of the material, allowing for measurable TS and WA values. In contrast, the TS of the MDF-like fiberboards could not be measured. Interestingly, lower molding temperatures were observed to reduce water resistance, as shown in Table 2, where fiberboards manufactured at a molding temperature of 160 °C could not maintain their cohesion in water. This indicates that the thermopressing conditions were inadequate to maintain material cohesion when exposed to moisture. Fiberboards are composed of lignocellulose material, which contains hydroxyl (-OH) groups. These groups form hydrogen bonds with water molecules, leading to increased water absorption and thickness swelling. This adversely affects the usability and applications of the boards in humid environments, as the moisture buildup in the fiber cell walls causes dimensional changes in the material [22,37].

When comparing the water sensitivity of coriander fiberboards thermopressed with the RocTool system to other coriander materials from a previous study [11], it is evident that the results are significantly worse here. This is likely due to the absence of waterproofing post-treatment, which can typically include surface coating with hydrophobic liquids (e.g., linseed oil, commercial varnish, etc.) [38] or thermal treatment. In particular, applying a thermal treatment at 200 °C to coriander fiberboards can reduce TS by 50% [11]. The heat treatment induces increasing mobility of polymeric chains and results in higher reactivity of lignins and proteins, favoring crosslinking reactions [35,39]. At the same time, the evaporation of water molecules allows for new hydrogen bonds to form between the proteins and cellulose hydroxyl groups [11]. This enhances the internal bonding of the boards and also reduces their hygroscopicity, thus decreasing moisture uptake. Therefore, for future work, it is recommended that coriander fiberboards originating from the RocTool system undergo thermal treatment to improve water resistance, making them more suitable for subsequent commercial applications.

According to ISO 16895:2016 [36], the manufactured coriander board with maximal flexural strength is considered a high-density fiberboard (HDF) as its nominal density is greater than 800 kg/m^3^. With a flexural strength, a flexural modulus of elasticity, and an internal bond strength of more than 38 MPa, 3.8 GPa, and 0.9 MPa, respectively, it is classified as a general-purpose high-density fiberboard for use in dry conditions (i.e., HDF-GP REG board). Thus, it is suitable for applications that may or may not require specific load-bearing properties, thereby broadening its potential uses. These include furniture, partition walls, veneer, and other construction materials. Oppositely, the manufactured MDF-like board does not yet fully meet the standard requirements for MDF for general purposes (i.e., 21 MPa flexural strength and 0.6 MPa internal bond strength). Minor adjustments in the thermopressing conditions can increase these mechanical properties. This would allow for it to meet the standard, and be considered a viable alternative to MDFs.

Nevertheless, it must be noted that the density of all the manufactured fiberboards (Table 2 and Table 4) is always high (1122 kg/m^3^ min) and much higher than that of commercial MDF (650–800 kg/m^3^). This increased density could hinder their implementation in the sector due to the challenges associated with transporting, maneuvering, and handling heavy materials on-site. However, it is important to recognize that higher density results in enhanced mechanical properties because particles are forced into closer proximity, thereby increasing surface contact [40]. Furthermore, it is observed that the high thermopressing conditions applied to obtain the fiberboard with maximal flexural strength produce a coriander fiberboard that is around 11% heavier than the MDF-like board (Table 4). Decreasing the thermopressing conditions for obtaining the MDF-like board not only benefits energy consumption and production costs but also reduces density, which could help address future challenges in transporting and maneuvering fiberboards.

To assess the environmental impact associated with the manufacturing of coriander fiberboards, a “gate-to-gate” life cycle assessment was carried out. Figure 3 shows the isoresponse curves for climate change (kg CO_2 eq_/m^2^) at mold temperatures of (a) 160 °C, (b) 180 °C, and (c) 200 °C, respectively. Figure 3a shows that increasing pressure at a 160 °C mold temperature does not lead to a significant increase in climate change impact, whereas the opposite effect is observed with increasing molding time, which notably accelerates the climate change indicator. This is in perfect accordance with the b_1_ coefficient of the associated polynomial model that is significantly lower than the b_2_ one (i.e., 0.4 and 1.1, respectively) (Table 3). Consequently, longer molding times result in higher CO_2 eq_ emissions, and the same trend is still observed at a 180 °C mold temperature in Figure 3b, and at a 200 °C mold temperature in Figure 3c. This effect is even more pronounced when combined with higher molding temperature, as the environmental indicator increases, indicating greater resource consumption and a larger environmental impact.

The results showed that under maximum thermopressing conditions (applied pressure: 35 MPa; molding time: 300 s; mold temperature: 205 °C), the coriander fiberboard production emitted 3.87 kg of CO_2 eq_ per m^2^, whereas under thermopressing conditions allowing for the production of an MDF-like board (applied pressure: 32.5 MPa; molding time: 170 s; mold temperature: 160 °C), the emissions were only 1.45 kg CO_2 eq_ per m^2^. This indicates that operating under such reduced thermopressing conditions lowers carbon emissions by 62.5%, highlighting the substantial decrease in resource consumption. It is important to highlight the isoresponse curves of Figure 3, as they identify thermopressing conditions that minimize environmental emissions and can help reduce the carbon footprint. This figure provides precise data for making informed decisions about sustainable production practices, promoting a balance between production efficiency and environmental responsibility. In summary, an isoresponse curve focused on predicting climate change in production is a valuable tool for enhancing environmental sustainability, optimizing processes, and complying with climate change regulations. In this case, the figure was constructed to study the impact of coriander fiberboard production on climate change. However, its potential is even greater, as it can be extended to other impact categories (such as ozone depletion, acidification, ecotoxicity, eutrophication, etc.) depending on the study’s objectives.

Additionally, it is important to highlight that besides their environmental benefits, coriander fiberboards are manufactured using a natural binder (i.e., the proteic-based deoiled coriander press cake), which eliminates the need for chemical adhesives that emit toxic components like VOCs (e.g., formaldehyde in the case of urea-formaldehyde (UF) or phenol–formaldehyde (PF) thermosetting resins) for obtaining cohesive materials, thereby preserving air quality. A recent study [11] confirmed that commercial MDF and especially chipboard emit a significant amount of carbonyl compounds at 23 °C, with formaldehyde representing 57% of these emissions, whereas the emission of carbonyl compounds was much less significant for the coriander-based board (−53% and −76%, respectively, in comparison with MDF and chipboard). In particular, among the three materials studied, coriander fiberboard was the only one with formaldehyde emissions below the detection limit, acetaldehyde representing the main contribution (90%) of the emitted carbonyl compounds. Thus, coriander panels are low-VOC, environmentally friendly materials, making them an excellent choice for construction. They contribute to better indoor air quality, reduce health risks, and have a low environmental impact.

## 4. Conclusions

In this study, 100% coriander-based fiberboards were successfully manufactured using thermopressing, combining refined straw and 40% (*w*/*w*) deoiled press cake. The primary goal was to optimize the production process through the use of an induction RocTool system. This study also examined the effects of key thermopressing parameters (i.e., applied pressure, molding time, and mold temperature) on the physical properties of the fiberboards, including density, flexural properties, surface hardness, and color. Additionally, cost and environmental impacts, particularly climate change, were evaluated.

Two optimal sets of thermopressing conditions were identified. The first set, designed to maximize flexural strength, involved an applied pressure of 35 MPa, a molding time of 300 s, and a mold temperature of 205 °C, producing a flexural strength of 40.6 MPa. The second set aimed to reduce energy consumption while producing fiberboards with properties comparable to commercial MDF panels. This set used an applied pressure of 32.5 MPa, a molding time of 170 s, and a mold temperature of 160 °C, yielding a flexural strength of 17.9 MPa, close to the target of 20.7 MPa, indicating that further refinement of the mathematical model is needed.

In terms of environmental impact, this study revealed that fiberboards produced under the maximum thermopressing conditions emitted 3.87 kg CO_2 eq_ per m^2^, while those produced under the MDF-like conditions reduced emissions to 1.45 kg CO_2 eq_, effectively halving carbon emissions. These findings highlight the potential of coriander-based fiberboards as a sustainable alternative to traditional wood-based boards, offering a reduced impact on climate change while avoiding the use of toxic fossil-based chemicals, such as formaldehyde-based thermosetting resins.

## Figures and Tables

**Table 1:** Experimental matrix of the thermopressing conditions using the RocTool system.

Board Number	*x*_1_	Applied Pressure (MPa)	*x*_2_	Molding Time (s)	*x*_3_	Mold Temperature (°C)
1	1.0	50	0.000	180	0.000	180
2	−1.0	10	0.000	180	0.000	180
3	0.5	40	0.866	284	0.000	180
4	−0.5	20	−0.866	76	0.000	180
5	0.5	40	−0.866	76	0.000	180
6	−0.5	20	0.866	284	0.000	180
7	0.5	40	0.289	215	0.816	200
8	−0.5	20	−0.289	145	−0.816	160
9	0.5	40	−0.289	145	−0.816	160
10	0.0	30	0.577	249	−0.816	160
11	−0.5	20	0.289	215	0.816	200
12	0.0	30	−0.577	111	0.816	200
13	0.0	30	0.000	180	0.000	180
14	0.0	30	0.000	180	0.000	180
15	0.0	30	0.000	180	0.000	180
16	0.0	30	0.000	180	0.000	180

**Table 2:** Characteristics, cost-related factors, and environmental indicator of the coriander fiberboards produced from the experimental design.

	Board 1	Board 2	Board 3	Board 4	Board 5	Board 6	Board 7	Board 8	Board 9	Board 10	Board 11	Board 12	Board 13	Board 14	Board 15	Board 16
Density (kg/m^3^)	1396 ± 18	1122 ± 64	1378 ± 18	1271 ± 27	1365 ± 26	1424 ± 30	1434 ± 31	1302 ± 35	1335 ± 22	1350 ± 52	1364 ± 29	1378 ± 32	1383 ± 28	1373 ± 47	1340 ± 46	1354 ± 49
Flexural properties																
*F_max_* (N)	36.9 ± 2.7	13.2 ± 0.4	42.1 ± 1.5	22.1 ± 1.6	29.4 ± 1.7	44.6 ± 3.7	46.2 ± 2.8	20.8 ± 2.9	25.8 ± 3.1	34.3 ± 2.9	44.6 ± 1.8	40.7 ± 0.5	39.0 ± 2.9	40.7 ± 2.0	40.7 ± 2.6	38.8 ± 1.7
*σ_f_* (MPa)	28.3 ± 1.1	6.6 ± 0.2	30.2 ± 1.0	14.8 ± 0.8	21.5 ± 1.2	34.8 ± 0.7	36.0 ± 1.6	14.3 ± 1.0	18.1 ± 0.4	24.1 ± 1.3	31.2 ± 1.1	31.3 ± 1.0	28.4 ± 1.2	30.5 ± 1.3	29.0 ± 1.1	28.1 ± 1.2
*E_f_* (GPa)	5.3 ± 0.3	1.0 ± 0.1	5.5 ± 0.2	2.2 ± 0.3	3.5 ± 0.3	6.2 ± 0.5	6.0 ± 0.2	2.3 ± 0.1	3.0 ± 0.2	4.5 ± 0.4	4.9 ± 0.2	5.4 ± 0.2	4.9 ± 0.4	5.3 ± 0.5	4.7 ± 0.3	4.8 ± 0.5
Surface hardness																
Shore D (°)	77.3 ± 1.9	65.4 ± 3.7	77.4 ± 2.0	71.2 ± 4.6	75.9 ± 3.2	78.4 ± 1.3	78.0 ± 1.4	72.9 ± 3.2	75.0 ± 2.7	77.0 ± 1.9	76.7 ± 2.4	75.5 ± 2.5	75.9 ± 2.3	76.5 ± 2.2	75.6 ± 1.9	75.1 ± 2.4
Color characteristics																
L*	70.8 ± 2.5	79.9 ± 4.6	70.0 ± 0.5	79.5 ± 1.1	74.6 ± 6.1	72.1 ± 0.2	71.0 ± 1.2	79.0 ± 0.4	75.7 ± 5.3	74.9 ± 1.2	73.2 ± 0.1	73.5 ± 4.2	73.0 ± 2.3	73.9 ± 2.1	73.9 ± 1.7	73.7 ± 2.2
a*	2.4 ± 0.7	4.3 ± 0.4	2.3 ± 0.1	4.1 ± 0.2	3.0 ± 1.3	2.8 ± 0.0	2.4 ± 0.4	3.9 ± 0.2	3.1 ± 1.2	3.1 ± 0.4	2.7 ± 0.0	2.4 ± 1.0	2.6 ± 0.8	2.7 ± 0.6	2.8 ± 0.6	2.8 ± 0.7
b*	3.9 ± 1.8	10.9 ± 3.0	3.5 ± 0.2	9.7 ± 0.8	6.1 ± 4.4	4.7 ± 0.2	3.7 ± 0.9	9.2 ± 0.3	6.5 ± 3.9	6.2 ± 0.9	5.1 ± 0.1	4.8 ± 3.1	5.0 ± 2.1	5.4 ± 1.6	5.6 ± 1.4	5.5 ± 1.8
Δ*E*	14.2 ± 3.1	4.1 ± 3.5	15.2 ± 0.5	4.0 ± 1.2	10.0 ± 7.5	12.7 ± 0.3	14.3 ± 1.6	4.7 ± 0.5	9.1 ± 6.5	9.7 ± 1.5	11.7 ± 0.2	11.8 ± 5.3	12.0 ± 3.2	11.0 ± 2.7	10.8 ± 2.2	11.0 ± 2.9
Water sensitivity																
TS (%)	98 *^1^*	n.m.	236 ± 19	n.m.	n.m.	232 ± 9	165 ± 37	n.m.	n.m.	n.m.	130 ± 14	55 *^1^*	n.m.	111 *^1^*	87 *^1^*	98 ± 12
WA (%)	180 ± 17	247 ± 17	170 ± 15	200 ± 8	193 ± 4	144 ± 10	78 ± 8	149 ± 14	163 ± 11	136 ± 7	146 ± 19	141 ± 10	131 ± 5	157 ± 13	166 ± 13	139 ± 13
Cost-related factors																
Energy consumption (kW h/m^2^)	28.6	17.4	40.8	8.6	10.9	31.9	33.0	14.9	19.4	29.4	26.3	15.3	23.0	23.0	23.0	23.0
Total manufacturing cost (€/m^2^)	8.55	5.74	11.58	3.53	4.12	9.37	9.64	5.10	6.23	8.73	7.96	5.21	7.15	7.15	7.15	7.15
Environmental indicator																
Climate change (kg CO_2 eq_/m^2^)	2.00	1.22	2.85	0.60	0.76	2.23	2.31	1.04	1.36	2.06	1.84	1.07	1.61	1.61	1.61	1.61

*^1^* No standard deviation given, as thickness swelling could only be measured on one of the three test specimens; n.m., non-measurable after 24 h immersion in water.

**Table 3:** Coefficients and R^2^ values for the quadratic polynomial models of the characteristics of the coriander fiberboards produced from the experimental design.

Coefficient	b_0_	b_1_	b_2_	b_3_	b_11_	b_22_	b_33_	b_12_	b_13_	b_23_	R^2^
Density (kg/m^3^)	1363	88	43	38	−103	31	15	−81	51	40	0.854
Flexural properties											
*F_max_* (N)	39.81	7.37	9.89	10.32	−14.79	−2.09	−2.41	−5.70	−0.08	−0.03	0.942
*σ_f_* (MPa)	29.0	6.8	7.7	8.6	−11.5	−1.0	−1.6	−6.5	2.9	−0.2	0.921
*E_f_* (GPa)	4.9	1.4	1.6	1.3	−1.8	−0.2	−0.4	−1.2	0.6	−0.7	0.911
Surface hardness											
Shore D (°)	75.8	3.9	2.6	1.1	−4.4	1.4	0.9	−3.3	0.7	1.2	0.879
Color characteristics											
L*	73.63	−3.83	−3.17	−2.42	1.72	0.02	0.93	1.56	0.19	0.17	0.971
a*	2.74	−0.79	−0.46	−0.53	0.69	0.23	0.09	0.32	0.22	0.21	0.948
b*	5.38	−2.85	−1.95	−1.70	2.03	0.12	0.25	1.35	0.36	0.19	0.963
Δ*E*	11.2	4.5	3.6	2.9	−2.0	−0.3	−0.9	−2.0	−0.4	−0.5	0.974
Cost-related factors											
Energy consumption (kW h/m^2^)	23.00	5.60	15.36	2.22	0.00	0.07	0.06	3.81	0.00	1.51	1.000
Total manufacturing cost (€/m^2^)	7.150	1.404	3.840	0.561	−0.005	0.002	−0.007	0.935	0.006	0.374	1.000
Environmental Indicator											
Climate change (kg CO_2 eq_/m^2^)	1.610	0.391	1.075	0.155	0.000	0.000	0.005	0.266	−0.002	0.103	1.000

**Table 4:** Characteristics of the coriander fiberboard optimized for maximal flexural strength (i.e., applied pressure: 35 MPa; molding time: 300 s; mold temperature: 205 °C), and of the MDF-like fiberboard produced from thermopressing conditions associated with minimal energy consumption (i.e., applied pressure: 32.5 MPa, molding time: 170 s, mold temperature: 160 °C), and comparison with values calculated from the mathematical models.

	Thermopressing Conditions for Maximal Flexural Strength		MDF-Like Thermopressing Conditions Associated with Minimal Energy Consumption	
Flexural Properties	Calculated from Mathematical Model	Obtained from the Experiment	Calculated from Mathematical Model	Obtained from the Experiment
Density (kg/m^3^)	1537	1458 ± 11	1346	1313 ± 11
F_max_ (N)	55	53.4 ± 3.3	29.7	28.0 ± 0.4
σ_f_ (MPa)	42.5	40.6 ± 1.7	20.7	17.9 ± 0.6
E_f_ (GPa)	7.4	6.7 ± 0.5	3.5	2.9 ± 0.2
Water sensitivity				
TS (%)	n.c.	161 ± 2	n.c.	n.m.
WA (%)	n.c.	123 ± 18	n.c.	198 ± 5
Internal bond strength (MPa)	-	1.45 ± 0.14	-	0.35 ± 0.07

n.c., non-calculable (non-existent mathematical model); n.m., non-measurable after 24 h immersion in water.

## Data Availability

The original contributions presented in the study are included in the article, further inquiries can be directed to the corresponding authors.
